# Proximity ligation assays of protein and RNA interactions in the male-specific lethal complex on *Drosophila melanogaster* polytene chromosomes

**DOI:** 10.1007/s00412-015-0509-x

**Published:** 2015-02-19

**Authors:** Henrik Lindehell, Maria Kim, Jan Larsson

**Affiliations:** Department of Molecular Biology, Umeå University, 90187 Umeå, Sweden

**Keywords:** Protein interaction, Dosage compensation, Polytene chromosomes, MSL complex

## Abstract

In *Drosophila*, the male-specific lethal (MSL) complex specifically targets the male X chromosome and participates in a twofold increase in expression output leading to functional dosage compensation. The complex includes five proteins and two non-coding RNAs (ncRNAs). A number of additional associated factors have also been identified. However, the components’ roles and interactions have not been fully elucidated. The in situ proximity ligation assay (PLA) provides a sensitive means to determine whether proteins and other factors have bound to chromosomes in close proximity to each other, and thus may interact. Thus, we modified, tested, and applied the assay to probe interactions of MSL complex components on polytene chromosomes. We show that in situ PLA can detect and map both protein-protein and protein-ncRNA interactions on polytene chromosomes at high resolution. We further show that all five protein components of the MSL complex are in close proximity to each other, and the ncRNAs *roX1* and *roX2* bind the complex in close proximity to MLE. Our results also indicate that JIL1, a histone H3 Ser10 kinase enriched on the male X chromosome, interacts with MSL1 and MSL2, but not MSL3 of the MSL complex. In addition, we corroborate proposed interactions of the MSL complex with both CLAMP and TopoII.

**Electronic supplementary material:**

The online version of this article (doi:10.1007/s00412-015-0509-x) contains supplementary material, which is available to authorized users.

## Introduction

Major collaborative projects including modENCODE (Celniker et al. 2009) and ENCODE (Consortium 2004), together with efforts of various groups (e.g., Filion et al. 2010), are providing vast sets of valuable high-resolution mapping data. To complement these resources, it is essential to identify histone modifications and binding sites of expression-regulating proteins and non-coding RNAs (ncRNAs) that are sufficiently close to confirm and map putative physical interactions between them. Chromatin immunoprecipitation (ChIP) mapping techniques have been highly useful for this purpose, but they typically display average binding patterns of factors in millions of cells. Thus, detected correlations in patterns may be due to antagonistic binding, e.g., two proteins binding at the same sites, but in different cells rather than binding in close proximity to each other.

In *Drosophila melanogaster* research, the endoreplicated polytene chromosomes (usually from third instar larval salivary gland cells) have long been used for mapping, high-quality assembly, and annotation of the species’ genome (Adams et al. 2000; Painter 1933, 1934). The amplification provided by the ~2000 tightly aligned chromatids also provides a powerful chromatin template for mapping associated factors at high resolution (10–50 kb) using immunostaining techniques (Lavrov et al. 2004). In addition, the polytene chromosomes are potentially ideal material for applications of a new technique to visually detect factors that bind close to each other, indicative of physical interaction: the in situ proximity ligation assay (Soderberg et al. 2006, 2008), hereafter in situ PLA. The technique involves use of two secondary antibodies with attached oligonucleotides (PLA probes). When these two probes are in close proximity, they can hybridize to a pair of connector oligonucleotides to form a complete circle after ligation. The spacing required for formation of a functional circle can be adjusted by varying the length of the oligonucleotides, but typically ranges between 28 and 40 nm. After ligation, one of the oligonucleotides acts as a primer for rolling circle amplification (Fire and Xu 1995), which can be visualized by a fluorescent probe.

In situ PLA of factors bound to polytene chromosomes is a potentially powerful strategy for probing not only interactions among associated proteins and ncRNAs but also the genomic sites of such interactions at high resolution. Thus, as both a proof-of-principle test and to acquire potentially valuable information, we have applied the technique to analyze interactions among polytene chromosome-bound components of the male-specific lethal (MSL) complex.

In fruit flies, the twofold difference in “dosage” of X chromosome genes in males and females (and between the X chromosome and autosomes in males) is compensated by a twofold increase in expression of genes on the single male X chromosome (Oliver 2007; Prestel et al. 2010a; Stenberg and Larsson 2011; Vicoso and Bachtrog 2009). The twofold increase in males results from a combination of a general buffering effect exerted on monosomic regions or chromosomes (Lundberg et al. 2012; Stenberg et al. 2009; Zhang et al. 2010) and an increase in expression from the male X chromosome mediated by the MSL complex (Deng et al. 2009; Hamada et al. 2005; Prestel et al. 2010a; Stenberg and Larsson 2011). The MSL complex consists of five MSL proteins (MSL1, MSL2, MSL3, MLE, and MOF) and two partly redundant non-coding RNAs, *roX1* and *roX2*. The complex binds most expressed genes on the male X chromosome, and MOF mediates acetylation of H4 at lysine 16 (H4K16ac). The resulting enrichment of H4K16ac on the male X chromosome is believed to cause de-condensation of the chromatin fiber, which at least partly explains the increased transcription output of this chromosome (Gelbart and Kuroda 2009; Philip and Stenberg 2013; Prestel et al. 2010a). The MSL complex not only tethers MOF to the male X chromosome but also limits its activation potential (Prestel et al. 2010b; Sun et al. 2013). Binding of the MSL complex to the X chromosome is thought to be initiated by sequence-dependent targeting to 100–200 nucleation sites termed chromatin entry sites (CES) or high-affinity sites (HAS) (Alekseyenko et al. 2008; Straub et al. 2008, 2013). This is followed by spreading to neighboring genes, via a process dependent on active transcription (Larschan et al. 2007; Sass et al. 2003), MSL complex concentration (Dahlsveen et al. 2006), affinity level (Lucchesi 2009; Straub et al. 2008), and sequence composition (Philip et al. 2012). Although the MSL complex mainly refers to the entire ribonucleoprotein complex, it has recently been suggested that partial MSL complexes with different constitutions and affinities for different chromatin interfaces are linked to HAS, promoters, and gene bodies (Straub et al. 2013). The MSL complex has been extensively mapped on the male X chromosome, and its chromosome associations clearly involve both protein-protein and protein-ncRNA interactions, which have been only partially elucidated. Thus, it seemed an ideal candidate for our test application of the in situ PLA technique to probe interactions of factors on polytene chromosomes.

We show here that in situ PLA is a sensitive, high-resolution technique for detecting and mapping protein-protein and protein-ncRNA interactions on polytene chromosomes. We also show that at the resolution provided by polytene chromosomes all five protein components of the MSL complex are in close proximity to each other, and the ncRNAs *roX1* and *roX2* bind the complex in close proximity to MLE. Our results also indicate that JIL1, a histone H3 Ser10 kinase enriched on the male X chromosome, interacts with MSL1 and MSL2 but not MSL3. In addition, we confirm proposed interactions between the MSL complex and both CLAMP and TopoII.

## Material and methods

### Polytene chromosome preparations

Flies were cultivated and crossed in vials containing potato mash-yeast-agar. We used the Oregon R strain as wild type, and *w; P[w* ^*+*^ *hsp83:msl2] msl3/ TM6B* females (from stock kindly provided by Mitzi Kuroda) to express MSL2 in an *msl3* mutant background in order to visualize high-affinity sites staining on polytene chromosomes (Dahlsveen et al. 2006; Demakova et al. 2003; Kelley et al. 1995) and compare their patterns to PLA staining patterns. Polytene chromosomes from the salivary glands of the third instar larvae were prepared essentially as previously described (Johansson et al. 2012; Lundberg et al. 2013). Briefly, salivary glands were dissected and fixed in 3.7 % formaldehyde in PBS, 0.3 % Triton X-100, for 40 s, followed by 2–3 min in 50 % acetic acid containing 1 % formaldehyde. Polytene chromosomes were squashed with high pressure using a MTC-200-1 precision vice (Penn Tool: Maplewood, NJ) as previously described by Novikov et al. (2007). The slides were quick-frozen in liquid nitrogen; the coverslip was removed; and the slides were stored in ethanol at −20 °C until required for use. Just before antibody incubation or in situ hybridization, the slides were air-dried and the areas with chromosome spreads were encircled using an ImmEdge Pen (Vector Laboratories).

### Primary antibody verification and proximity ligation assays

We verified that the primary antibodies to be used in the analyses (Table S1) could recognize targets bound to polytene chromosomes and function appropriately in the in situ PLA as follows. The air-dried slides were rehydrated in phosphate-buffered saline with 0.1 % Triton X-100 (PBT) for 30 min, transferred to blocking solution (0.1 M maleic acid, 0.15 M NaCl, 1 % Boehringer blocking reagent), and incubated for 30 min at room temperature. Primary antibodies to be tested were added (singly, diluted in 20 μl of blocking solution), and the resulting mixtures were each covered by a cover slip and incubated overnight at 4 °C. The slides were then washed 2× for 5 min in a solution containing 0.1 M maleic acid, 0.15 M NaCl, and 0.3 % Tween 20 (pH 7.5). A 60 μl drop of PLA probe mixture was added, and the slides were incubated (open) in a humidity chamber at 37 °C for 1 h.

A PLA probe mixture consists of two PLA probes (labeled secondary antibodies), designated PLUS and MINUS, raised against the species of the primary antibodies. Commercial PLA probes (Olink Biosciences) were routinely used at 1:5 dilution, and custom-made probes—e.g., donkey anti-rat antibodies from Jackson ImmunoResearch, labeled using a Duolink in situ Probemaker Minus kit (Olink Biosciences)—at 1:500 dilution. Following the initial incubation after adding the probes, the slides were washed 2× for 5 min with a solution containing 0.1 M maleic acid, 0.15 M NaCl, and 0.3 % Tween 20 (pH 7.5). Excess washing solution was removed, and the slides were incubated with 60 μl of ligation mixture in an open drop in a humidity chamber at 37 °C for 1 h, according to instructions supplied with the Duolink in situ Orange Starter Kit (Olink Biosciences). Following ligation, the slides were washed 2× for 2 min in a solution containing 10 mM Tris–HCl, 150 mM NaCl, 0.05 % Tween 20 (pH 7.4). Excess washing solution was tapped off, and the slides were incubated with 60 μl of amplification mixture in an open drop in a humidity chamber at 37 °C for 100 min in darkness.

The amplification mixture includes all reagents needed for the rolling circle amplification and fluorophores that hybridize with the amplified product. The amplification mixture was prepared according to the supplier’s instructions (Olink Biosciences). All following steps were performed in dim light. After amplification, the slides were washed 2× for 5 min in a solution containing 200 mM Tris–HCl, 100 mM NaCl (pH 7.5) then once for 1 min in 2 mM Tris–HCl, 1 mM NaCl (pH 7.5). The slides were air-dried in the dark and mounted in Duolink in situ Mounting Medium with DAPI (Olink Biosciences) and incubated at 4 °C overnight to allow DAPI to stain the chromosomes. Alternatively, 0.5 mg/ml DAPI was added to the PLA probe mixture, allowing direct microscopic analysis. Preparations were examined using a Zeiss Axiophot microscope equipped with a KAPPA DX20C charge-coupled device camera. Images were assembled and digitally merged using Adobe Photoshop.

### Protein-protein interaction assay

To probe interactions between pairs of the focal proteins (and methylation sites), we used mixtures of corresponding pairs of primary antibodies (individually verified as described above), raised in different organisms. Portions (25 μl) of the diluted primary antibody solutions were added to the polytene chromosome preparations, which were then incubated overnight at 4 °C. The following steps were as described above, except that the two PLA probes were against the two different species of the primary antibodies.

### RNA-protein interaction assay

To determine whether the ncRNAs *roX1* and *roX2* bound in close proximity to any of the focal proteins on the prepared polytene chromosomes, the air-dried slides were rehydrated in an ethanol series: 1 min each in 95, 70, and 30 % ethanol followed by 15-min incubation in PBT. The slides were next incubated for 15 min in PBT, 3.7 % formaldehyde, then washed 3× 3 min in PBT. The slides were then incubated in hybridization solution (5× SSC, 5× Denhardts solution, 500 μg/ml herring sperm DNA, 250 μg/ml yeast tRNA, 50 % formamide) for 4 h in a humidity chamber.

For hybridization, an antisense *roX1* or *roX2* RNA probe was synthesized and labeled with biotin (Roche, 11685597910) using a full-length *roX2* cDNA clone (*GH18991*) or *roX1* (*GH10432*) and SP6 RNA polymerase (Roche). The probe was mixed in hybridization solution to a final concentration of 2.5 μg/ml, denatured for 5 min at 65 °C, chilled on ice and reheated to 42 °C then added to the preparations on the slides. The slides were sealed by a coverslip and rubber cement, then hybridized overnight at 42 °C. Following hybridization, the slides were washed once for 10 min in 2× SSC at room temperature, followed by 2× 20 min in 5× SSC, 50 % formaldehyde, 10 mM dithiothreitol at 42 °C; 2× 30 min in 2× SSC at 42 °C; once at 60 min in 0.1× SSC at room temperature; and finally 10 min in PBT at room temperature. After washing, the slides were incubated for 30 min in blocking solution (0.1 M maleic acid, 0.15 M NaCl, 1 % Boehringer blocking reagent). The primary antibodies (against biotin and the protein partner to be tested) diluted in blocking solution were then added, and the mixture was incubated overnight at 4 °C. The following washing and PLA reactions were performed as outlined above.

### Analyses of protein-protein and *roX2*-protein interactions by the proximity ligation assay combined with immunostaining

To combine PLA detection of interactions with immunostaining of one of the probed proteins a secondary antibody coupled to AlexaFluor 488 was added to the PLA probe mixture. For example, to visualize JIL1-MSL1 interaction together with immunostaining, the primary antibodies used were JIL1 (rabbit) and anti-MSL1 (rat). In the next step, the PLA mixture contained anti-Rat PLA Minus, anti-Rabbit PLA Plus (as described above), and Donkey anti-rabbit conjugated with AlexaFluor 488 diluted 1:500 (Molecular Probes). When secondary antibodies are used as PLA probes as in this study, the maximum distance for interaction is estimated to approximately 35 nm (Olink Biosciences).

## Results

### Validation of antibodies for use in the proximity ligation assay

**Fig. 1:**
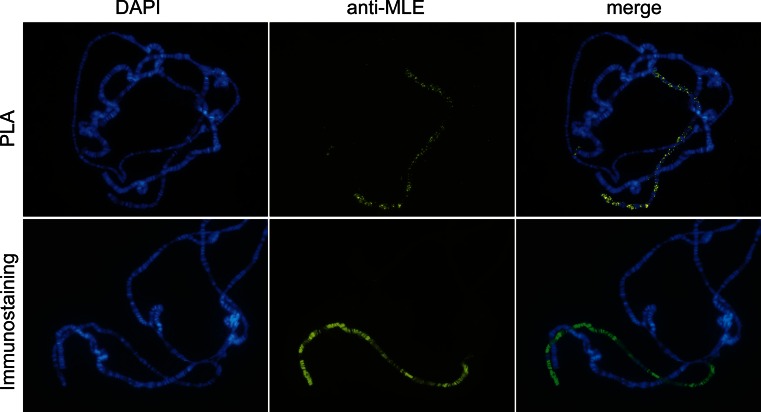
The in situ proximity ligation assay (PLA) technique reproduces the immunostaining-based MLE binding pattern. Polytene chromosomes from wild-type third instar larvae immunostained using the in situ PLA (*top row*) and traditional immunostaining with a secondary antibody coupled to Alexa Fluor 488 (*bottom row*). The PLA signals are clearly more punctuated, and their intensity varies substantially less than the immunostaining signals

A primary objective was to test the ability of the in situ PLA to detect pairs of proteins in close proximity when bound to polytene chromosomes, since this would give information on both potential interactions among them and their genomic binding sites. As a first step, we verified that all antibodies to be used could function correctly in the assay by incubating them singly with the polytene chromosome spreads then adding corresponding pairs of Plus and Minus PLA probes. As shown in Fig. 1, the binding patterns typically obtained match traditional immunostaining patterns perfectly in terms of overall morphology. However, the PLA signals have a smaller intensity range and are punctuated, the number of dots reflecting the extent of interaction.

### The MSL complex proteins interact when targeted on the X chromosome

**Fig. 2:**
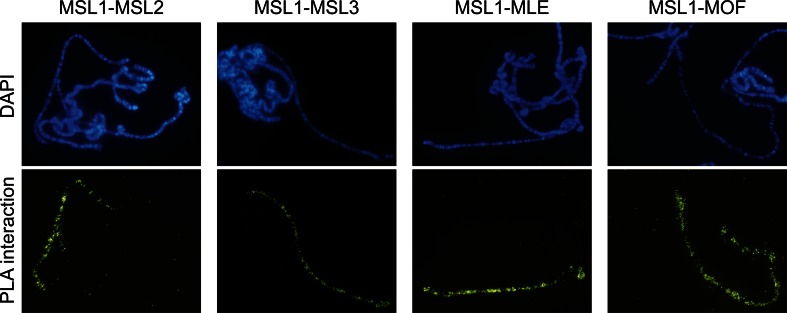
In situ PLA verifies close proximity between all members of the MSL complex, as illustrated here by signals obtained using combinations of probes for MSL1 and (*from left to right*) MSL2, MSL3, MLE, and MOF. All of these combinations yield strong signals along the male X chromosome

Several studies have detected high degrees of colocalization of MSL complex components using immunostaining and chromatin immunoprecipitation techniques (Kelley et al. 1995; Kind et al. 2008; Lyman et al. 1997; Straub et al. 2013). Our two other major objectives were to evaluate the ability of the in situ PLA to determine if the components bind sufficiently closely to interact physically, and if so, improve the resolution of their binding patterns. For this purpose, we first applied pairwise tests of all MSL complex components (Table 1). The results corroborate the interactions of the tested components, as the close proximity of component pairs were visualized as clear enrichment of signals on the X chromosome (Fig. 2, Suppl. Fig. 1). If only one primary antibody was added no enrichment was detected on chromosomes (Suppl. Fig. 1).

**Table 1:** Summary of potential in situ proximity ligation assay-detected interactions

Interactors	MSL1 rt	MSL2 gt	MSL3 rt	MSL3 gt	MLE rt	MOF rt	*roX1 mo*	*roX2* mo
MSL1 rb	^NT^	+	+	+	^NT^	^NT^	^NT^	^NT^
MSL2 rb	+	^NT^	+	+	^NT^	+	^NT^	^NT^
MLE rb	+	+^a^	+	+	^NT^	^NT^	+	+
MOF rb	+	+^a^	+	+	+	^NT^	^NT^	^NT^
JIL1 rb	+	+^a^	^NT^	−	^NT^	^NT^	^NT^	^NT^
CLAMP rb	^NT^	+^a^	^NT^	+	+	^NT^	^NT^	^NT^
TopoII rb	+^b^	−	−	−	+	^NT^	^NT^	^NT^
H3K36me3 rb	^NT^	^NT^	−	−	^NT^	^NT^	^NT^	^NT^

*rb* rabbit, *rt* rat, *gt* goat, *mo* mouse, *+* interacts with enrichment on the male X chromosome, *−* no detected enrichment, *NT* not tested

^a^Weak enrichment on the male X chromosome probably caused by the quality of the MSL2 goat antibody

^b^Weak enrichment of MSL1-TopoII as compared to the strong signal of TopoII-MLE

**Fig. 3:**
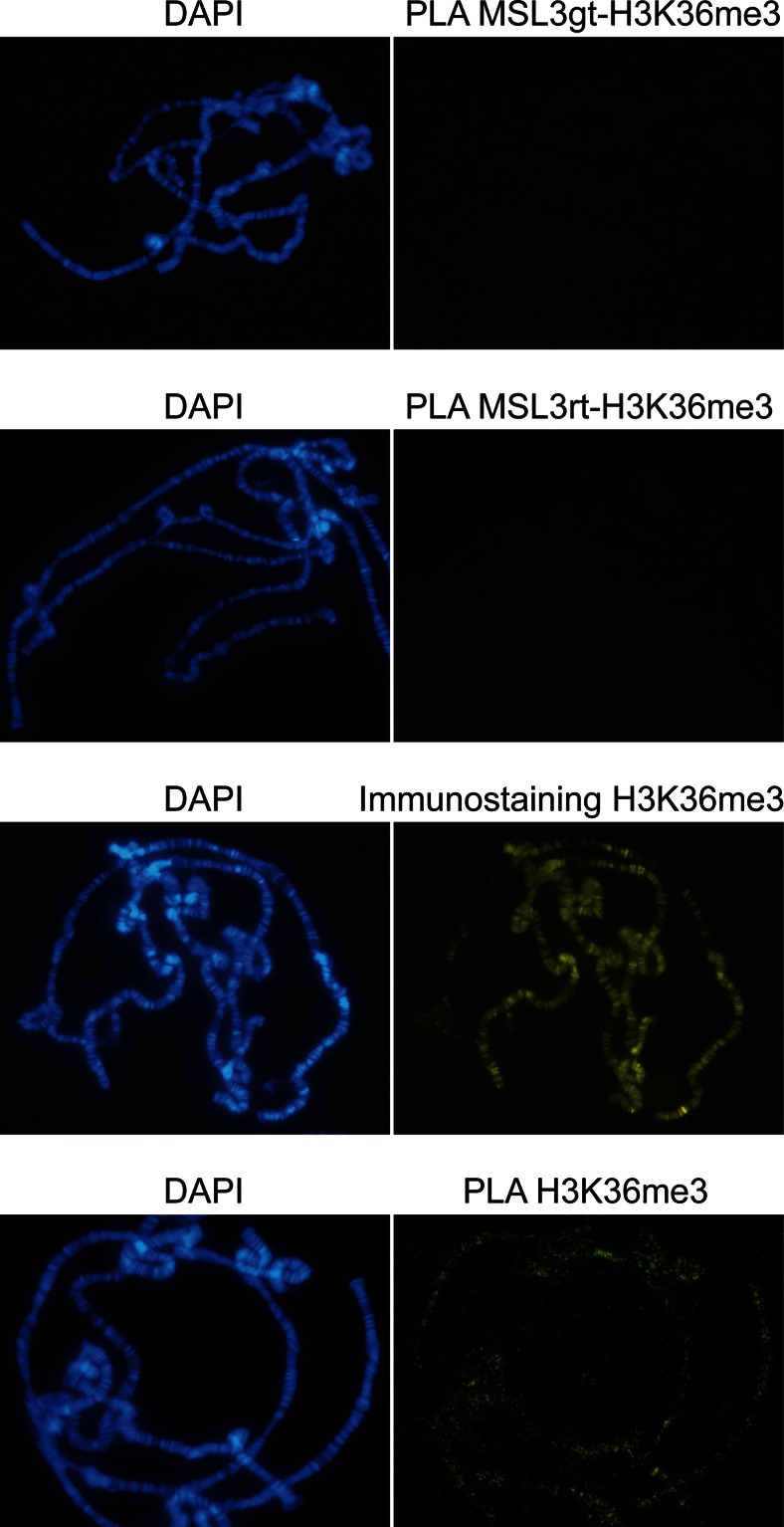
Colocalization of two factors is not enough to result in a PLA signal. No enrichment on the male X chromosome is seen when using in situ PLA to probe proximity between MSL3 and H3K36me3 (*first and second rows*). Immunostaining (*third row*) as well as anti-H3K36me3 primary antibody verification (*fourth row*) result in robust enrichment on chromosomes

Since all MSL complex components interacted and considering the rolling circle amplification step, we next asked if background targeting could result in a PLA signal enriched on one of the two components individual targets. We also tested the sensitivity of the method, i.e., if it can distinguish between proteins in a complex from colocalizing factors. First, we tested if strong binding of two antibodies at different sites could lead to background PLA signals from these discrete targets. For this purpose, we tested effects of using a strong rabbit antibody against the chromosome 4-specific protein Painting of Fourth (Johansson et al. 2007; Larsson et al. 2004) and a strong rat antibody against MSL1 (Mendjan et al. 2006). Using this combination, no signal enrichment on either the male X chromosome or the fourth chromosome was detected (Suppl. Fig. 2). Next, we tested the ability of antibodies directed against MSL complex components and other factors that colocalize according to immunostaining and ChIP-chip analysis, but probably less closely, to generate in situ PLA signals. For this test, we used antibodies against the histone modification H3K36me3 and MSL3. H3K36me3 is enriched in bodies of active genes (Kharchenko et al. 2011), like the MSL complex (Alekseyenko et al. 2006; Gilfillan et al. 2006), and suggested to have role in the recruitment and spread of the MSL complex via the chromo-domain of MSL3 (Larschan et al. 2007). Even though H3K36me3 is enriched on chromosomes and detected both by immunostaining and PLA, no interaction between MSL3 and H3K36me3 was detected using the PLA technique (Fig. 3, Table 1). We conclude that in situ PLA is a sensitive and specific technique for detecting proteins bound to polytene chromosomes in sufficiently close proximity for physical interactions between them, including all components of the MSL complex.

### *roX* ncRNA interacts with the MSL complex as revealed by PLA

**Fig. 4:**
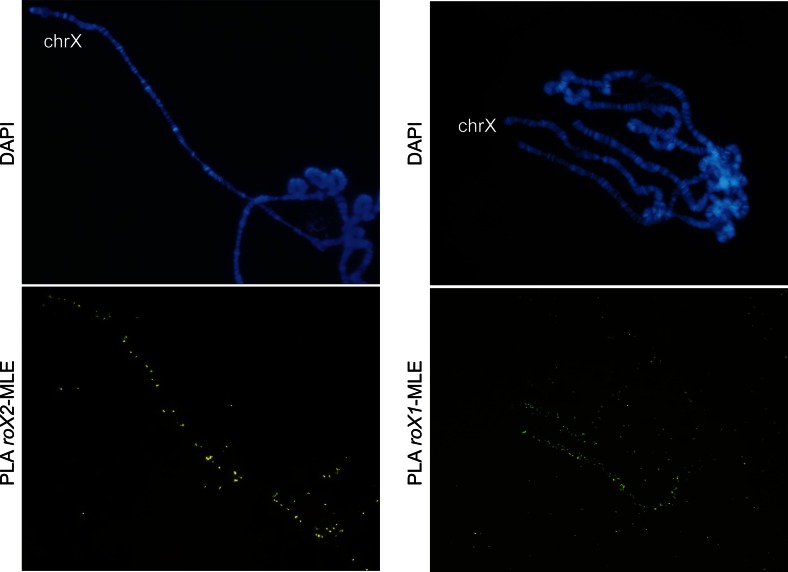
The ncRNAs *roX1* and *roX2* interact with MLE along the male X chromosome. In situ PLA detected close proximity between *roX2* and MLE (*left column*); *roX1* and MLE (*right column*) using biotin-labeled anti-sense RNA probes with primary antibodies against biotin and MLE

In addition to the five MSL proteins, the MSL complex includes at least one of the two non-coding RNAs *roX1* and *roX2*. Thus, we also tested the ability of in situ PLA to detect proximity between ncRNAs and proteins on polytene chromosomes, using biotin-labeled antisense *roX1* and *roX2* RNA and antibodies against biotin and MLE. This resulted in clear enrichment of PLA signals on the male X chromosome (Fig. 4), verifying that MLE interacts with *roX1* and *roX2* and demonstrating that in situ PLA can detect ncRNA-protein interactions on polytene chromosomes.

### PLA combined with immunostaining reveals interactions between JIL1 and MSL1 or MSL2 but not MSL3

**Fig. 5:**
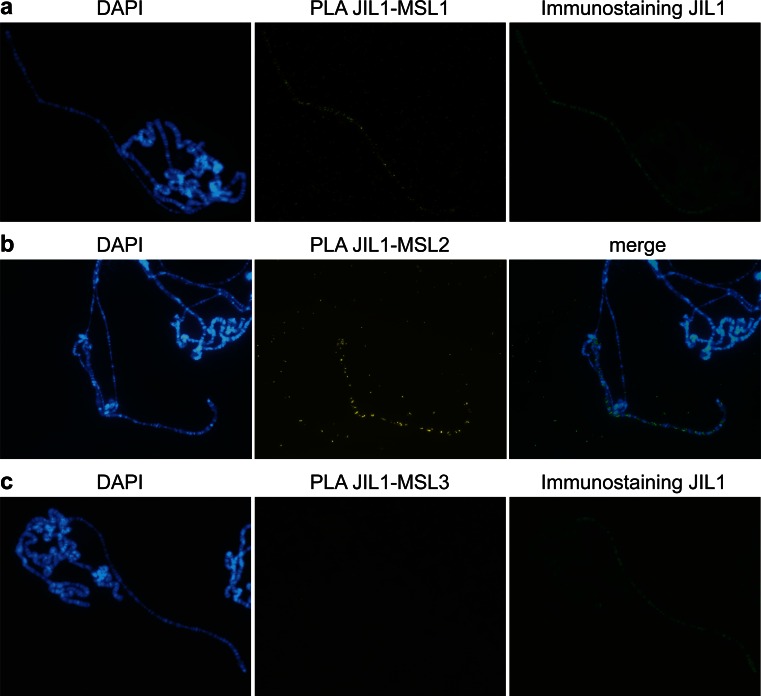
JIL1 interacts with MSL1 and MSL2 along the male X chromosome. Polytene chromosomes from wild-type third instar larvae stained using PLA JIL1-MSL1 combined with traditional immunostaining (**a**). The *left*, *middle*, and *right panels* show a nucleus stained by DAPI, in situ PLA results, and results of traditional immunostaining with a secondary antibody coupled to Alexa Fluor 488, respectively. PLA JIL1-MSL2 shows interaction along the male X chromosome (**b**) in contrast to no detected interaction for PLA JIL1-MSL3 (**c**)

Having verified the ability of the in situ PLA to detect potential protein-protein interactions, we then tested proposed, but unverified, interactions and combined the technique with traditional immunostaining using a fluorochrome providing distinct signals. We started by testing the potential interaction between JIL1 and the MSL complex. Use of JIL1 and MSL1 or MSL2 (but not MSL3) probes resulted in clear enrichment of signals from the male X chromosome (Fig. 5, Table 1). Further, the in situ PLA results for the JIL1 and MSL1 or MSL2 combinations provide a punctuated, but otherwise identical, form of the JIL1 immunostaining pattern on the male X chromosome (Fig. 5). We conclude that although JIL1 and the MSL complex colocalize all along the male X chromosome, only MSL1 and MSL2 bind sufficiently close to JIL1 for in situ PLA proximity detection.

### The MSL complex interacts with CLAMP and TopoII

**Fig. 6:**
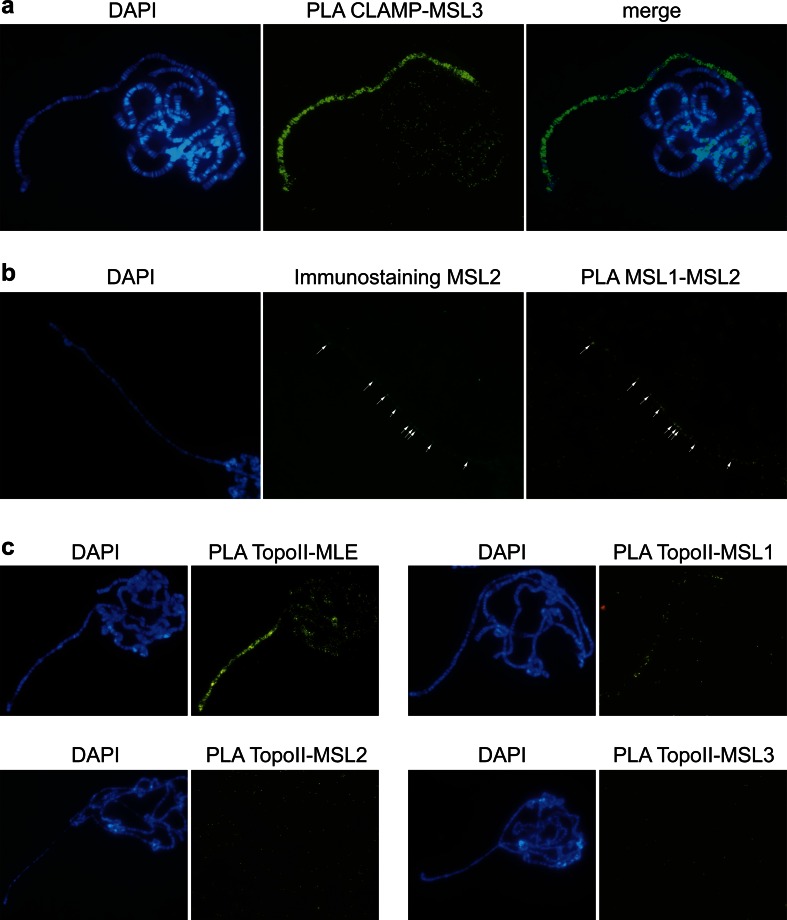
CLAMP and TopoII interactions with the MSL complex are verified by in situ PLA. **a** Strongly enriched signals along the male X chromosome indicating that CLAMP and MSL3 interaction is not restricted to high-affinity sites. **b** High-affinity sites visualized by immunostaining and PLA MSL1-MSL2 on polytene chromosomes from *w; P[w* ^*+*^ *hsp83:msl2] msl3/ TM6B* females expressing MSL2 in an *msl3* mutant background. *Arrows* indicate some of the high-affinity sites along the X chromosome. **c** In situ PLA signals providing indications of close proximity along the male X chromosome between TopoII and MLE (*top left panel*), weak but detectable signal suggestive of proximity between TopoII and MSL1 (*top right panel*), and no detectable interaction between TopoII and either MSL2 or MSL3 (*left and right bottom panels*)

Recently, additional interactions have been claimed for the MSL complex, such as interaction with the Zinc finger protein CLAMP at high-affinity sites (Soruco et al. 2013; Soruco and Larschan 2014; Wang et al. 2013), and type II topoisomerase (TopoII) via MLE (Cugusi et al. 2013). We tested these two proposed interactions and verified that CLAMP binds sufficiently close to the MSL complex for interaction. However, comparison of the in situ PLA signals to the observed binding patterns of MSL components at high-affinity sites visualized by expression of MSL2 in *msl3* mutant females (*w; P[w* ^*+*^ *hsp83:msl2] msl3/ TM6B*) indicated that the interaction is not restricted to high-affinity sites as proposed, but rather extends to all binding sites of the MSL complex on the male X chromosome (Fig. 6). Our in situ PLA results also corroborate the proposed interaction between MLE and TopoII (Fig. 6). Intriguingly, the combination of TopoII and MLE probes yielded strong signals from the male X chromosome, MSL1 and TopoII probes yielded much weaker signals, and both MSL3-TopoII and MSL2-TopoII probe combinations yielded no apparent signals (Fig. 6). The results suggest that MLE and MSL1 bind in close proximity to TopoII, but MSL2 and MSL3 bind beyond the interaction range.

## Discussion

Large-scale international projects have produced highly detailed catalogues of genomic functional elements in humans (ENCODE project) and various model organisms (modENCODE). Correlations between positions of these elements and putatively associated factors’ colocalization patterns are commonly used as predictors of interactions. Here, we show that the in situ proximity ligation assay provides a novel approach for detecting pairs of factors in close proximity on the endoreplicated *Drosophila* polytene chromosomes. Thus, it provides sensitive indications of whether factors do (or do not) physically interact and the genomic sites of their interactions. The results show that in situ PLA can be successfully applied to probe interactions on polytene chromosomes and reproduces conventional immunostaining patterns. Further, since the in situ PLA technique used here depends on a rolling circle amplification, it results in a punctuated staining pattern, in which each dot represents one interaction site and the number of dots reflects the extent of interaction. However, since the use of two antibodies that yield high background signals could theoretically generate a shared in situ PLA background response, we recommend verification of the suitability of all candidate antibodies using the single antibody PLA test strategy outlined above as well as conventional immunostaining. Promisingly, when the technique is applied to large structures, such as polytene chromosomes, the background can be distinguished from signals of interest if it is not localized to chromosomes or does not coincide with binding sites determined by conventional immunostaining.

### All five MSL proteins may interact when bound to the X chromosome

The MSL complex refers to the five proteins MSL1, MSL2, MSL3, MOF, and MLE together with *roX1* and/or *roX2* ncRNAs. The four proteins MSL1, MSL2, MSL3, and MOF form a stable core complex (Alekseyenko et al. 2014; Mendjan et al. 2006; Morales et al. 2004; Smith et al. 2000; Wang et al. 2013), while MLE binding to the core is less stable, RNA-dependent when attached to chromosomes, and sensitive to extraction conditions applied during purification procedures (Akhtar et al. 2000; Richter et al. 1996; Smith et al. 2000). The prevailing model assumes that all MSL proteins are involved in binding of the complex to high-affinity sites and its spread (Conrad and Akhtar 2011; Gelbart and Kuroda 2009; Straub and Becker 2011), but this model has recently been challenged. Following the first ChIP mapping of MLE together with the use of a high-shear ChIP-seq technique, Straub et al. (2013) proposed that partial MSL complexes with differing compositions are associated with different chromatin interfaces. They predicted that chromatin contact is provided by MSL1 and MOF at promoters, MSL3 at gene bodies (via interaction with H3K36me3), and MSL2 together with MLE at high-affinity sites. Hence, the cited authors proposed that MSL complex architecture differs at these locations. Our in situ PLA analysis detected no qualitative differences among the pairwise interactions, contrary to expectations based on the proposed model. It should be stressed that potential differences in interaction patterns of the individual components between promoters and gene bodies would probably not be detected at the resolution provided by polytene chromosomes. However, our results conflict with the restriction of complete stable complexes to high-affinity sites implied by the proposed model. In fact, we detected clear enrichment on the X chromosome with all tested pairwise combinations, reproducing the immunostaining patterns of single MSL proteins. The observed differences in signal strength (number of dots), which could theoretically reflect transient contact, correlate with the strength of the primary antibodies used and cannot at this point be interpreted as differences in complex architecture.

### The proposed interaction between MSL3 and H3K36me3 is not confirmed by PLA

One model for the spreading of the MSL complex from high-affinity sites to active gene bodies suggests that interaction between the chromo-domain of MSL3 with H3K36me3 stabilizes the association between the complex and active genes (Larschan et al. 2007; Sural et al. 2008). The model is to some extent based on the correlation between the distribution of H3K36me3 sites and MSL complex binding patterns. We detected no PLA indications of close proximity between MSL3 and H3K36me3, but this may be due to transience of the putative interaction. It cannot be excluded that the lack of PLA signals reflects that H3K36me3 epitopes bound by MSL3 are occluded from detection by H3K36me3-specific antibodies, yet such epitopes remain available on the adjacent nucleosomes, which explains the positive PLA result when validating the HSK36me3 antibody. It should be stressed that although the interactions between chromo-domains and methylated histones are well documented, the affinity is relatively weak. For example, dissociation constants for both HP1a interaction with H3K9me and Polycomb chromo-domain interactions with H3K27me are in the micromolar range (Fischle et al. 2003; Jacobs and Khorasanizadeh 2002; Jacobs et al. 2001). Alternatively, the MSL3 chromo-domain may mainly interact with the H4K20 monomethyl mark, as suggested by structural studies (Kim et al. 2010). Regardless of which (if any) of these hypotheses are true, our results clearly indicate that in situ PLA provides higher resolution indications of the proximity of factors bound to polytene chromosomes than ChIP colocalization analysis.

### JIL1 interacts with the MSL complex via MSL1 and MSL2

JIL1, a histone H3 Ser10 kinase, is believed to counteract heterochromatin formation (Jin et al. 1999, 2000; Regnard et al. 2011) and is highly enriched on the male X chromosome. Correct targeting of JIL1 on the male X chromosome is genetically dependent on the MSL complex and indications that JIL1 is loosely attached to the MSL complex have already been published (Jin et al. 2000; Wang et al. 2013). *Inter alia*, a V5-tagged JIL1 fusion protein, has been shown to immunoprecipitate MSL1, MSL2, and MSL3 (Jin et al. 2000). In contrast, our in situ PLA results include indications that JIL1 interacts with MSL1 and MSL2, but not MSL3. However, S2 cells were used in the cited immunoprecipitation experiment and the results do not reflect solely interactions of complexes bound to chromosomes (unlike our in situ PLA results). Notably, the lack of JIL1-MSL3 interaction cannot be explained by antibody quality since both JIL1 and MSL3 are strong antibodies in all other experiments.

### CLAMP and TopoII interact with the MSL complex

In an attempt to identify additional factors that interact with the MSL complex, Kuroda and colleagues applied ChIP analysis followed by mass spectrometric analysis of formaldehyde cross-linked chromatin (Wang et al. 2013). Using MSL3 as bait, an expanded group of associated proteins was identified, including not only the core MSL complex proteins and JIL1 but also other proteins including TopoII and CLAMP. Interaction between CLAMP and the MSL complex has since been further supported (Soruco et al. 2013; Soruco and Larschan 2014). The proposed association with TopoII has been analyzed, and it has been suggested that the MSL complex recruits TopoII to the X chromosome via MLE (Cugusi et al. 2013).Accordingly, we detected strong PLA signals on the male X chromosome indicative of interaction between TopoII and MLE, weak signals indicative of interaction between TopoII and MSL1, but no indications of interaction between TopoII and either MSL2 or MSL3. The results are compatible with MLE binding in close proximity to TopoII, MSL1 at the limit of the interaction range, and both MSL2 and MSL3 binding outside the PLA-detectable interaction range. For CLAMP, we found indications of a close proximity with MSL3, but in contrast to the proposed interaction being restricted to high-affinity sites, we detected signals all along the X chromosome, reproducing the normal targeting of the MSL complex.

In summary, we demonstrate that in situ PLA is a valuable new addition to the *Drosophila* research toolbox as a sensitive method for detecting expression-regulating proteins and ncRNAs that bind to polytene chromosomes sufficiently closely for physical interaction. The method has high potential utility for both verifying proposed interactions and identifying their genomic sites.

## Electronic supplementary material

Below is the link to the electronic supplementary material.

ESM 1 (PDF 966 kb)
