# Supplementary material for: Proximity ligation assays of protein and RNA interactions in the male-specific lethal complex on Drosophila melanogaster polytene chromosomes
Source: Chromosoma. 2015 Feb 19;124(3):385–95. doi: 10.1007/s00412-015-0509-x (PMC4548014; doi:10.1007/s00412-015-0509-x)
Supplement: Supplementary file 1 — (PDF 966 kb) [file 412_2015_509_MOESM1_ESM.pdf]

Supplementary material for

Proximity ligation assays of protein and RNA interactions in the Male-specific lethal complex on *Drosophila melanogaster* polytene chromosomes

Henrik Lindehell[1], Maria Kim[1], and Jan Larsson[1*]

[1]Department of Molecular Biology, Umeå University, SE-90187 Umeå, Sweden

This PDF includes:

Supplementary Figures 1-2 and Supplementary Table 1

SUPPLEMENTARY FIGURE LEGENDS

Suppl. Fig. 1 Close proximity between individual members of the MSL-complex is verified by *in situ* PLA. Signals obtained using probes for all pairs of MSL-complex components are shown. The signal strengths correlate with the strengths of the primary antibody. In particular the relatively weak goat anti-MSL2 antibody (sc32458) used in combinations with MLE and MOF antibodies yields weaker signals than all of the other antibodies used. However, all combinations provide signal enrichment along the male X-chromosome. As a negative control, no specific enrichment is detected when only one primary antibody is used, e.g. anti-MSL2 (rat) as primary antibody and two PLA probes rabbit-PLUS and rat-MINUS (MSL2 only).

Suppl. Fig. 2 No detectable signal enrichment was generated when using probes for the chromosome 4-specific POF protein and the X-chromosome-specific MSL1 protein on either the male X-chromosome or the fourth chromosome.

Supplementary Table 1 Antibodies used in proximity ligation assay

Target	Species	Supplier	Reference
**MSL1**	rabbit	Mitzi Kuroda	
**MSL1**	rat	Asifa Akhtar	(Mendjan et al. 2006)
**MSL2**	rabbit	Mitzi Kuroda	(Kelley et al. 1995)
**MSL2**	goat	Santa Cruz, (dG-20): sc-32458	
**MSL3**	goat	Mitzi Kuroda	
**MSL3**	rat	Asifa Akhtar	(Mendjan et al. 2006)
**MLE**	rabbit	Mitzi Kuroda	(Palmer et al. 1994)
**MLE**	rat	Asifa Akhtar	(Mendjan et al. 2006)
**MOF**	rabbit	Asifa Akhtar	
**MOF**	rat	Asifa Akhtar	(Mendjan et al. 2006)
**biotin**	mouse	Jackson ImmunoResearch, (200-002-211)	
**JIL1**	rabbit	Peter Becker	(Regnard et al. 2011)
**Topoisomerase II**	rabbit	Paul Fisher	
**CLAMP**	rabbit	Erica Larschan	(Soruco et al. 2013)
**POF**	rabbit		(Larsson et al. 2004)
**H3K36me3**	rabbit	Abcam, ab9050	

Kelley RL, Solovyeva I, Lyman LM, Richman R, Solovyev V, Kuroda MI (1995) Expression of *msl-2* causes assembly of dosage compensation regulators on the X chromosomes and female lethality in *Drosophila*. Cell 81:867-877

Larsson J, Svensson MJ, Stenberg P, Mäkitalo M (2004) Painting of fourth in genus *Drosophila* suggests autosome-specific gene regulation. Proc Natl Acad Sci U S A 101:9728-9733

Mendjan S et al. (2006) Nuclear pore components are involved in the transcriptional regulation of dosage compensation in *Drosophila*. Mol Cell 21:811-823

Palmer MJ, Richman R, Richter L, Kuroda MI (1994) Sex-specific regulation of the male-specific lethal-1 dosage compensation gene in *Drosophila*. Genes Dev 8:698-706

Regnard C, Straub T, Mitterweger A, Dahlsveen IK, Fabian V, Becker PB (2011) Global analysis of the relationship between JIL-1 kinase and transcription. PLoS Genet 7:e1001327

Soruco MM et al. (2013) The CLAMP protein links the MSL complex to the X chromosome during *Drosophila* dosage compensation. Genes Dev 27:1551-1556

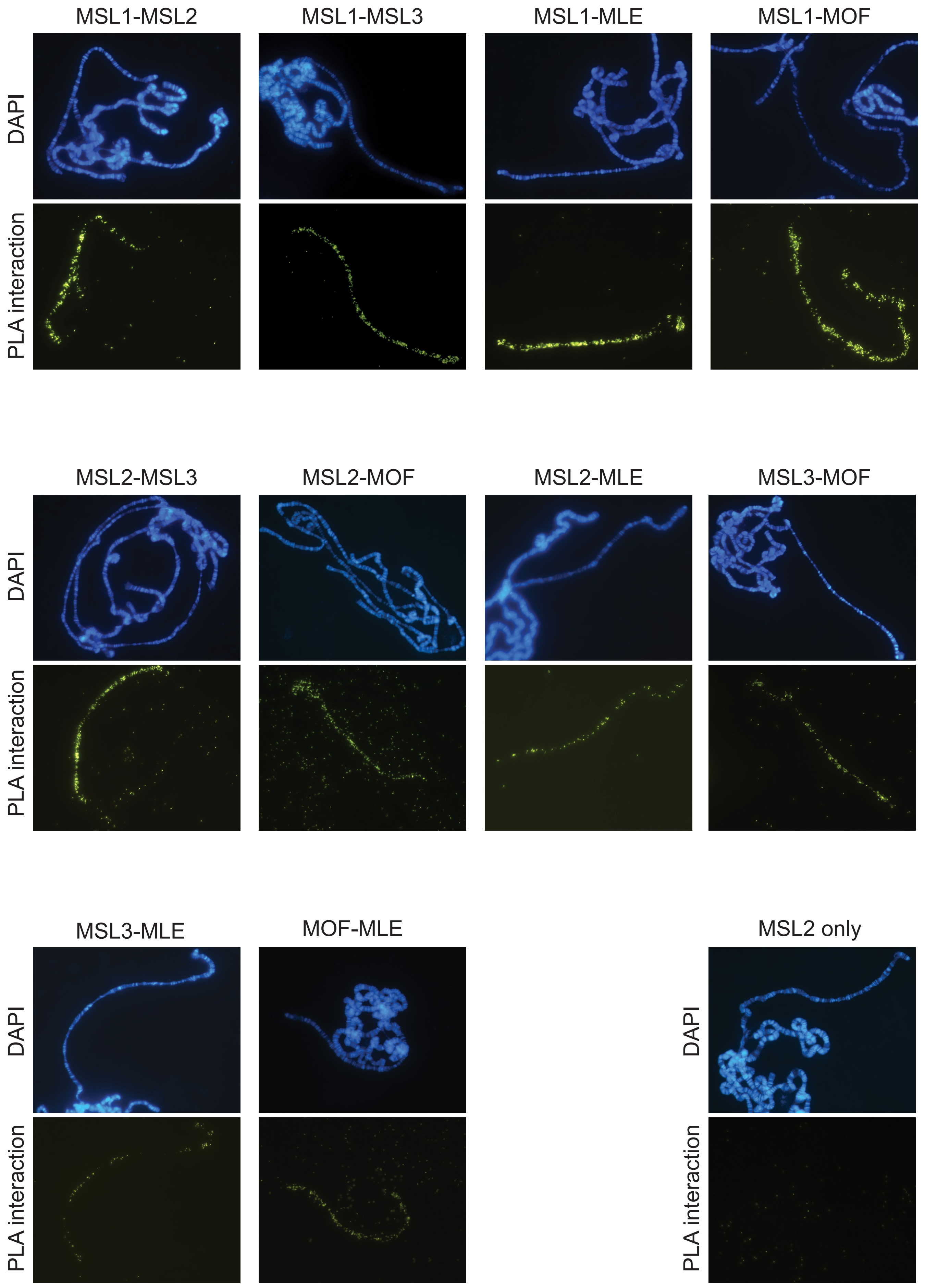
MSL1-MSL2
MSL1-MSL3
MSL1-MLE
MSL1-MOF
DAPI
PLA interaction
MSL2-MSL3
MSL2-MOF
MSL2-MLE
MSL3-MOF
DAPI
PLA interaction
MSL3-MLE
MOF-MLE
MSL2 only
DAPI
PLA interaction

Supplementary Figure 2

DAPI

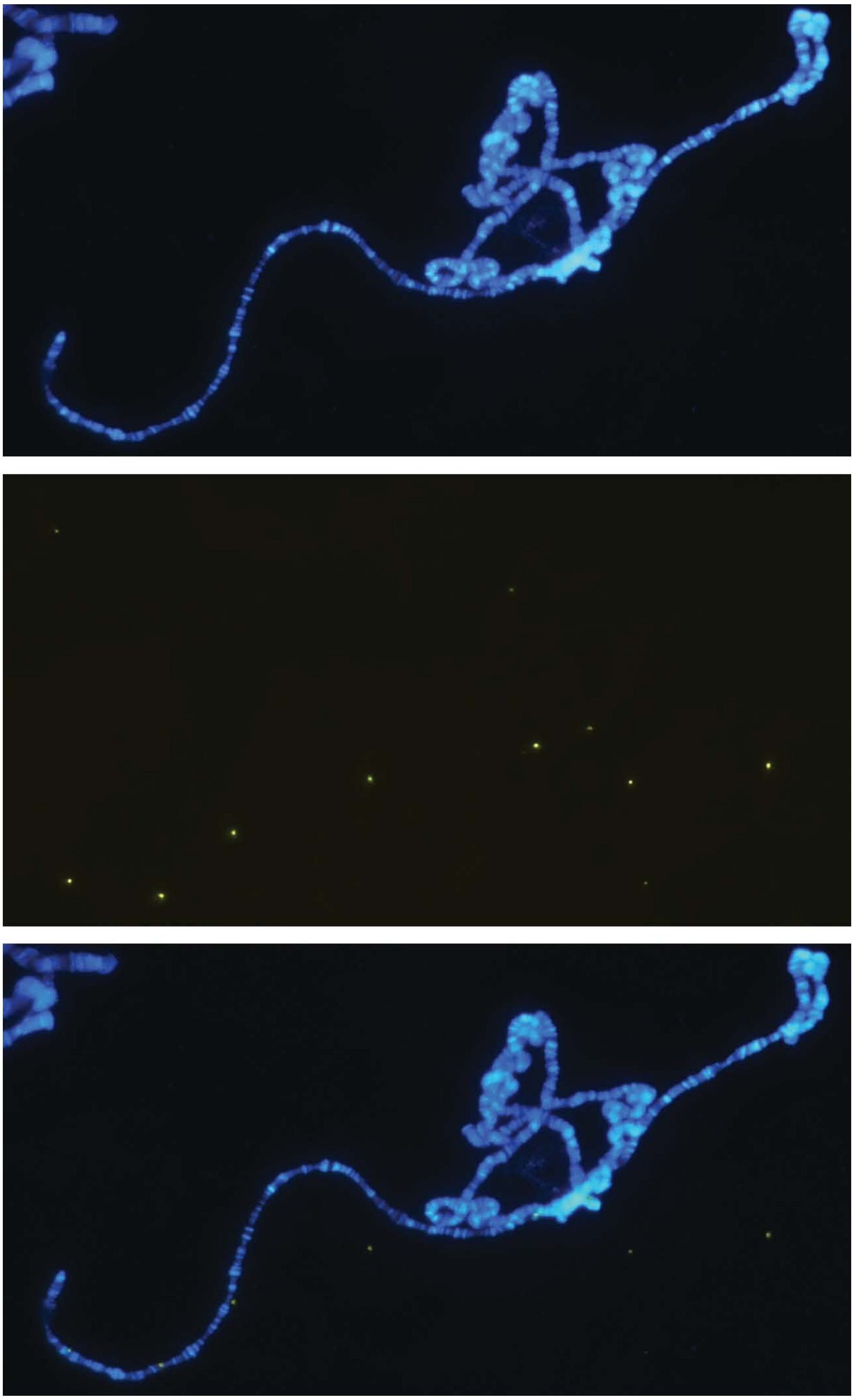

PLA POF-MSL1

merge
